# Novel Bacterial Topoisomerase Inhibitor Gepotidacin Demonstrates Absence of Fluoroquinolone-Like Arthropathy in Juvenile Rats

**DOI:** 10.1128/aac.00483-22

**Published:** 2022-10-18

**Authors:** Cindy Fishman, Jessica M. Caverly Rae, Lorraine M. Posobiec, Susan B. Laffan, Steven A. Lerman, Neil Pearson, Salim Janmohamed, Etienne Dumont, Deatra Nunn-Floyd, Dinesh J. Stanislaus

**Affiliations:** a Nonclinical Safety, GSK Research and Development, Collegeville, Pennsylvania, USA; b Medicinal Chemistry, GSK Research and Development, Collegeville, Pennsylvania, USA; c Clinical Sciences, GSK Research and Development, Brentford, Middlesex, United Kingdom; d Clinical Sciences, GSK Research and Development, Collegeville, Pennsylvania, USA

**Keywords:** gepotidacin, children, pediatric, antibiotic resistance, fluoroquinolone, arthropathy, juvenile rat, nonclinical study, preclinical study, drug safety, drug toxicity

## Abstract

Fluoroquinolone use in children is limited due to its potential toxicity and negative effects on skeletal development, but the actual effects/risks of fluoroquinolones on bone growth and the mechanisms behind fluoroquinolone-driven arthropathy remain unknown. Gepotidacin is a novel, bactericidal, first-in-class triazaacenaphthylene antibiotic with a unique mechanism of action that is not anticipated to have the same risks to bone growth as those of fluoroquinolones. Gepotidacin is in phase III clinical development for uncomplicated urinary tract infections (ClinicalTrials.gov identifiers NCT04020341 and NCT04187144) and urogenital gonorrhea (ClinicalTrials.gov identifier NCT04010539) in adults and adolescents ≥12 years of age. To inform arthropathy and other potential toxicity risks of gepotidacin in pediatric studies, this nonclinical study assessed oral gepotidacin toxicity in juvenile rats from postnatal day (PND) 4 to PND 32/35 (approximately equivalent to human ages from newborn to 11 years), using both in-life assessments (tolerability, toxicity, and toxicokinetics) and terminal assessments (necropsy with macroscopic and microscopic skeletal femoral head and/or stifle joint examinations). Gepotidacin doses of ≤300 mg/kg of body weight/day were well tolerated from PND 4 to PND 21, and higher doses of ≤1,250 mg/kg/day were well tolerated from PND 22 when the dose levels were escalated to maintain systemic exposure levels up to PND 35, with no observed treatment-related clinical signs, effects on mean body weight gain, or macroscopic findings on articular surfaces. A dose of 1,000 mg/kg/day was not tolerated during the dosing period from PND 4 to 21, with effects on body weight gain, fecal consistency, and body condition. Microscopic effects on articular surfaces were evaluated after 32 days of gepotidacin treatment at the highest tolerated dose. After 32 days of treatment with the highest tolerated gepotidacin dose of 300/1,250 mg/kg/day (systemic concentrations [area under the curve {AUC} values] of 93.7 μg · h/mL [males] and 121 μg · h/mL [females]), no skeletal effects on articular surfaces of the femoral head or stifle joint were observed. The absence of treatment-related clinical signs and arthropathy in juvenile rats provides evidence to support the potential future use of gepotidacin in children.

## INTRODUCTION

Quinolones are synthetic antibacterial agents with the basic chemical structure of a bicyclic ring. The addition of a fluorine atom at position C-6 and various substitutions on the basic quinolone structure created a new generation of so-called “fluoroquinolone” antibiotics (e.g., norfloxacin, ciprofloxacin, levofloxacin, and moxifloxacin) (1, 2). Quinolones and fluoroquinolones are highly effective antibacterial agents that work by inhibiting the synthesis of bacterial DNA. Specifically, they bind to intracellular bacterial topoisomerase enzymes, forming drug-enzyme-DNA complexes that prevent transcription and subsequent bacterial replication.

In clinical practice, fluoroquinolones are commonly used for the treatment of respiratory infections and urinary tract infections (UTIs) in adults (1); however, their use in children is limited due to safety concerns related to skeletal development. Following observations of age-related arthropathy in the weight-bearing joints of juvenile animals (3- to 12-month-old dogs [3–5], 7- and 12-week-old rabbits [5, 6], 7-day-old CF-1 mice [7], and 4-week-old rats [5]) receiving quinolones in preclinical studies, the U.S. Food and Drug Administration (FDA) advised against the use of fluoroquinolones in clinical trials involving patients under 18 years of age, and therefore, approved pediatric indications for fluoroquinolones are very limited (8). Additionally, the FDA and the European Medicines Agency (EMA) recommend restrictions on the use of fluoroquinolone and quinolone antibiotics in adults and children, and the FDA has approved fluroquinolone label changes (9) due to evidence of negative side effects, mostly involving muscles, bones, and the nervous system (10, 11); however, since there has been no evidence of significant irreversible musculoskeletal side effects resulting from fluoroquinolone use in children, the safety of fluoroquinolone use in growing children is debated (12–15).

Several potential mechanisms of toxicity for the observed fluoroquinolone arthropathy in juvenile animal models have been described. These include the inhibition of mitochondrial DNA synthesis in immature chondrocytes (4), fluoride-driven direct toxicity to cartilage (12), and a deficiency of magnesium in cartilage due to the magnesium-chelating properties of quinolones (16). However, the precise mechanism of quinolone-driven arthropathy remains unknown.

Gepotidacin is a novel, bactericidal, first-in-class triazaacenaphthylene antibiotic that inhibits bacterial DNA replication by a distinct mechanism of action (17, 18), which confers clinically relevant activity against most strains of target pathogens such as Escherichia coli, Staphylococcus saprophyticus, and Neisseria gonorrhoeae, including those resistant to current antibiotics (19–21). Gepotidacin is in phase III clinical development for the treatment of uncomplicated UTI (uUTI) in adult and adolescent females ≥12 years of age (ClinicalTrials.gov identifiers NCT04020341 and NCT04187144) (22, 23) and urogenital gonorrhea (GC) in adult and adolescent males and females ≥12 years of age (ClinicalTrials.gov identifier NCT04010539) (24). Furthermore, a pediatric investigation plan and a pediatric study plan have been agreed upon with the EMA and FDA, respectively (GlaxoSmithKline, unpublished data), to support the potential future use of gepotidacin for the treatment of uUTIs in children aged ≥2 years. Molecular structural analyses of gepotidacin demonstrated the selective inhibition of bacterial DNA gyrase and topoisomerase IV via a unique binding mechanism that is distinct from that of other quinolone antibiotics (17–19), including fluoroquinolones. Unlike fluoroquinolones, gepotidacin does not either contain a fluorine atom or bind to magnesium (18, 25) and has not been shown to engage other potential mechanisms associated with arthropathy.

In this nonclinical study to assess the tolerability and plasma toxicokinetics of gepotidacin in juvenile rats from postnatal day (PND) 4 to PND 32/35 (approximately equivalent to human ages from newborn to 11 years [26]), an additional objective was to inform the risk of arthropathy in future pediatric clinical studies in children 2 to 11 years of age. This study was performed in an animal model of an appropriate species and age range (the ages of the rats included in this study encompass this developmental period in humans), achieved gepotidacin exposure levels above clinical exposures (as detailed previously by Barth et al. [27]), and maintained a treatment duration sufficient to detect potential fluoroquinolone-like arthropathy in rodents (5, 7).

(Some of the material discussed in this article has been presented as a poster presentation at the European Society of Clinical Microbiology and Infectious Diseases-American Society for Microbiology Joint Conference on Drug Development, Dublin, Ireland, 4 to 7 October 2022 [28]).

## RESULTS

### Tolerability-and-toxicokinetics phase (round 1).

**FIG 1:**
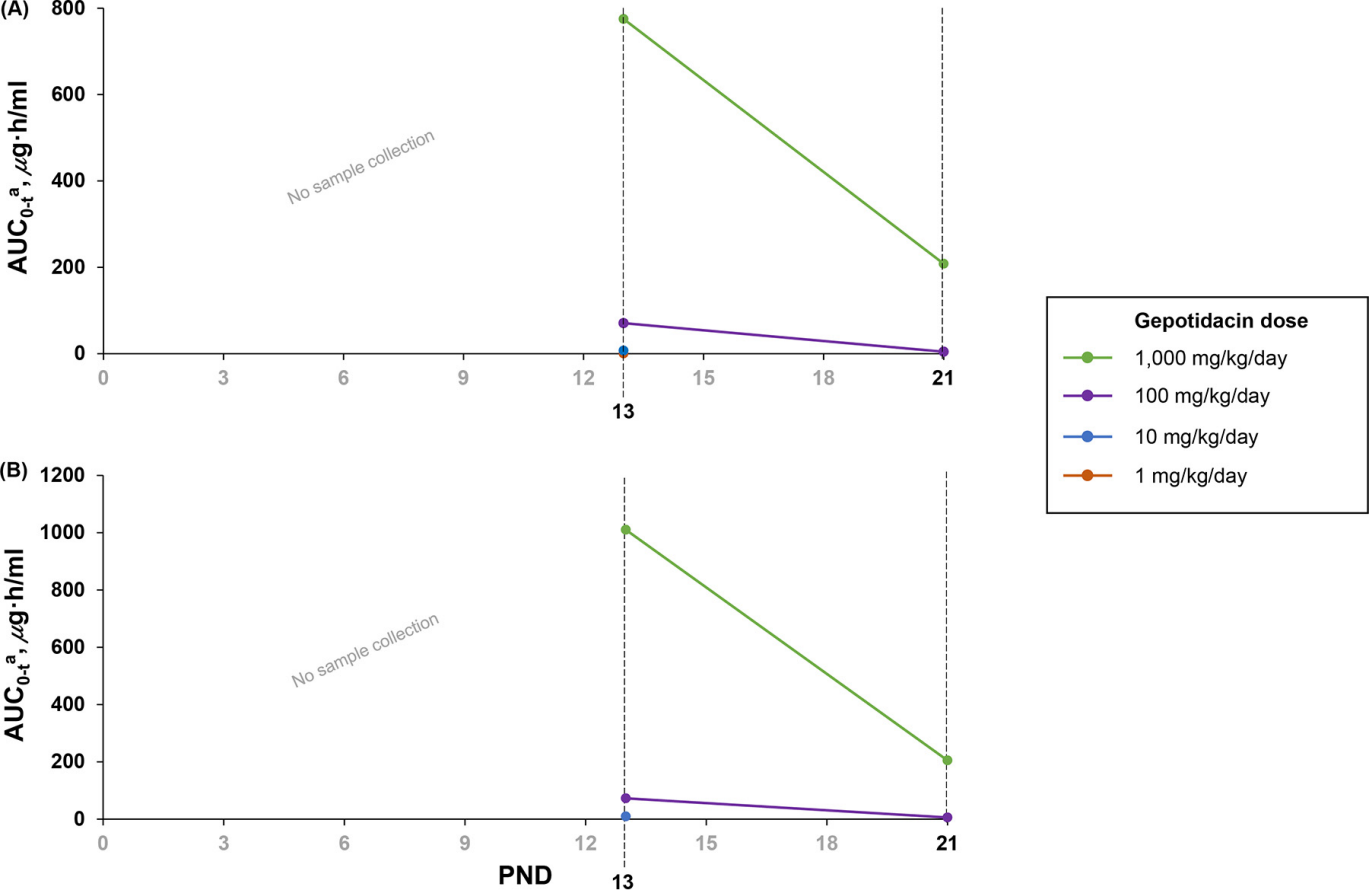
Round 1 mean area under the curve from time zero to the last measurable concentration (AUC_0–_*_t_*) values in males (A) and females (B) on postnatal day (PND) 13 and PND 21 by gepotidacin dose showing a marked decrease in exposure at PND 21 compared to PND 13. The days on which samples were collected are marked in black text. ^a^For the purpose of calculating the AUC_0–_*_t_*, the concentrations at time zero were assigned with the concentrations at 24 h. Female parameters for a 1-mg/kg/day gepotidacin dose on PND 13 and male and female parameters for 1- and 10-mg/kg/day gepotidacin doses on PND 21 could not be calculated due to limited measurable data.

Doses of 1, 10, 100, or 1,000 mg/kg of body weight/day were tolerated when given once daily from PND 4 to PND 21. There were no treatment-related clinical signs or effects on mean body weight gains for pups given 1, 10, or 100 mg/kg/day. At 1,000 mg/kg/day, loose feces were evident for the majority of male and female pups starting approximately 1 week after dosing, with decreases in mean body weight gain for male and female pups from PND 4 to PND 7, compared with the other treatment groups (data not shown). Large decreases in systemic exposure were evident in juvenile rats between PND 13 and PND 21 at both 100 and 1,000 mg/kg/day (round 1 area under curve from time zero to the last measurable concentration [AUC_0–_*_t_*] values are shown in Fig. 1; other exposure parameters are shown in Table S1 in the supplemental material).

### Toxicity-and-toxicokinetics phase (rounds 2 and 3).

**(i) Round 2.** In round 2, 4 groups were dosed by oral gavage (0, 30/300, 300/1,000, or 1,000/1,500 mg/kg/day for 20 pups/sex/group [5 litters/group], with the lower dose on PND 4 to PND 21 and the higher dose on PND 22 to PND 32/35). Between PND 5 and PND 9, a dose of 1,000 mg/kg/day in the highest-dose group unexpectedly caused an excessive number of treatment-related early deaths (5 males and 8 females from 4 litters, including dead or missing and presumed cannibalized pups). No prior clinical signs, effects on body weight, or visceral abnormalities were noted in the pups that died. The remaining juvenile rats in the 1,000-mg/kg/day group showed overall poor condition (loose feces and decreased body weight gains relative to the control group) from PND 4 to PND 21 (Table 1). Thus, due to excessive toxicity, all remaining juvenile rats in the highest-dose treatment group were euthanized on PND 21 before dose escalation.

**TABLE 1:** Round 2 mean absolute weight gains, by sex and gepotidacin dose^*a*^

Treatment and variable	Value for group											
	Males						Females					
	PND 4–7	PND 7–14	PND 14–21	PND 4–21	PND 21–28	PND 28–35	PND 4–7	PND 7–14	PND 14–21	PND 4–21	PND 21–28	PND 28–35
Vehicle control												
No. of pups	19	19	19	19	13	13	20	20	20	20	16	14
Mean (SEM) absolute wt gain (g)	5.66 (0.18)	16.38 (0.41)	18.74 (0.37)	40.78 (0.66)	44.49 (1.52)	54.22 (2.72)	5.58 (0.10)	15.98 (0.39)	17.46 (0.29)	39.01 (0.42)	42.17 (0.94)	45.39 (1.66)
Gepotidacin at 30/300 mg/kg/day^*b*^												
No. of pups	20	12	12	12	12	12	20	12	12	12	11	11
Mean (SEM) absolute wt gain (g)	6.38 (0.15)	18.75 (0.70)	21.78 (0.55)	47.03 (1.10)	44.53 (1.05)	57.63 (1.81)	6.06 (0.15)	18.38 (0.31)	20.93 (0.84)	45.32 (0.85)	39.00 (0.92)	42.99 (1.31)
Gepotidacin at 300/1,000 mg/kg/day^*b*^												
No. of pups	19	11	11	11	11	11	20	12	12	12	12	12
Mean (SEM) absolute wt gain (g)	5.44 (0.22)	18.93 (0.63)	24.86 (1.01)	49.28 (1.36)	44.75 (1.48)	58.48 (2.22)	4.99 (0.18)	18.29 (0.58)	22.43 (0.69)	45.88 (0.87)	38.55 (0.97)	46.23 (1.19)
Gepotidacin at 1,000/1,500 mg/kg/day^*b*^												
No. of pups	18	17	17	17	–	–	19	17	16	16	–	–
Mean (SEM) absolute wt gain (g)	2.50 (0.23)	14.69 (0.76)	18.51 (1.10)	35.70 (1.96)	–	–	2.74 (0.27)	15.20 (0.61)	19.61 (0.96)	37.88 (1.78)	–	–

a There were six deaths that were not determined to be treatment related: 2 pups were euthanized following blood collection-related trauma, 1 pup in the control group died from a partially torn trachea (dosing accident), and 3 pups from 1 litter were missing or partially cannibalized due to poor maternal behavior. A dash indicates that these data were not collected due to the early termination of the dose group.

b Dose escalation with the 1st dose from postnatal day (PND) 4 to PND 21 and the 2nd dose from PND 22 to PND 32/35.

There was no treatment-related effect on mortality or clinical signs for juvenile rats given ≤300/1,000 mg/kg/day; the mean body weight gains were up to 1.21 times higher than those of the vehicle control groups at doses of 30/300 and 300/1,000 mg/kg/day from PND 4 to PND 21 (Table 1).

**FIG 2:**
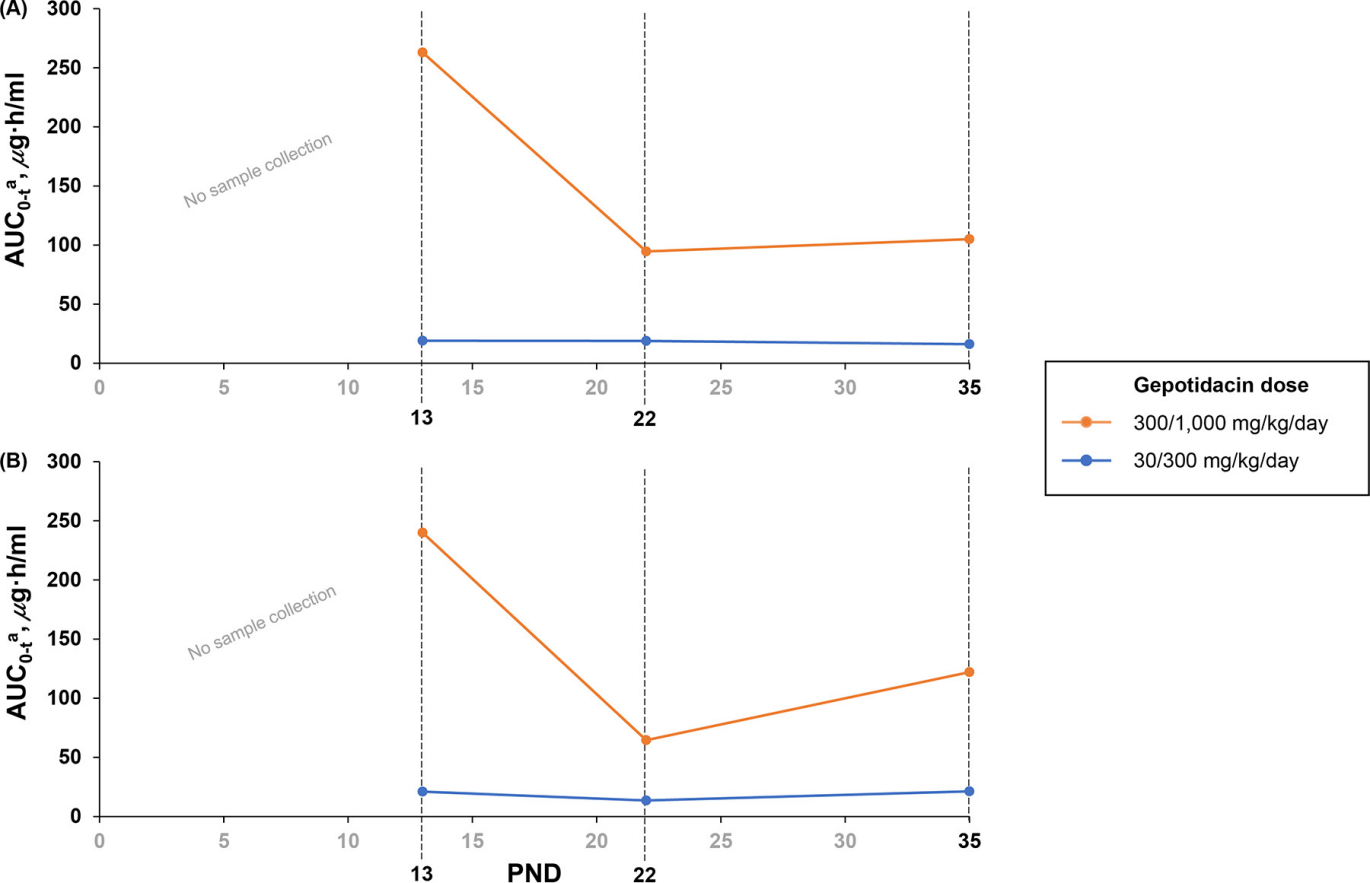
Round 2 mean area under the curve from time zero to the last measurable concentration (AUC_0–_*_t_*) values in males (A) and females (B) on postnatal day (PND) 13, PND 22, and PND 35 by gepotidacin dose with dose escalation on PND 22 to maintain systemic exposure as much as possible across the age range. The days on which samples were collected are marked in black text. ^a^For the purpose of calculating the AUC_0–_*_t_*, the concentrations at time zero were assigned with the concentrations at 24 h.

There was good separation for the AUC_0–_*_t_* and the maximum concentration of the drug (*C*_max_) (increases of 12.6-fold and 10.8-fold, respectively) between the 30/300- and 300/1,000-mg/kg/day dose levels, indicating adequate systemic exposure. Dose escalation was successful at 30/300 mg/kg/day; similar AUC values were observed across different postnatal ages during the dosing period. For 300/1,000 mg/kg/day, lower AUC_0–_*_t_* values (approximately 2- to 3.7-fold lower) were observed on PND 22 or PND 35 than on PND 13 (round 2 AUC_0–_*_t_* values are shown in Fig. 2; other exposure values are shown in Table S2).

**(ii) Round 3.** Doses of 100/1,000 and 300/1,250 mg/kg/day were given from PND 4 to PND 32 (with dose escalation on PND 22). There were no treatment-related clinical signs or effects on mean body weight gains for male and female pups given doses of 0, 100/1,000, or 300/1,250 mg/kg/day (Table 2).

**TABLE 2:** Round 3 mean absolute weight gains, by sex and gepotidacin dose^*a*^

Treatment and variable	Value for group									
	Males					Females				
	PND 4–7	PND 7–14	PND 14–21	PND 4–21	PND 21–28	PND 4–7	PND 7–14	PND 14–21	PND 4–21	PND 21–28
Vehicle control										
No. of pups	12	12	12	12	6	12	12	12	12	6
Mean (SEM) absolute wt gain (g)	6.88 (0.33)	19.74 (1.11)	23.68 (0.67)	50.30 (1.96)	42.92 (2.59)	6.63 (0.24)	18.53 (0.91)	22.30 (0.82)	47.46 (1.82)	37.88 (2.64)
Gepotidacin at 100/1,000 mg/kg/day^*b*^										
No. of pups	12	12	12	12	6	12	12	12	12	6
Mean (SEM) absolute wt gain (g)	6.74 (0.15)	16.89 (0.32)	24.43 (0.65)	48.06 (0.63)	43.95 (1.32)	5.95 (0.19)	15.88 (0.33)	22.20 (0.61)	44.03 (0.91)	39.40 (0.91)
Gepotidacin at 300/1,250 mg/kg/day^*b*^										
No. of pups	12	12	12	12	6	12	11	11	11	5
Mean (SEM) absolute wt gain (g)	6.85 (0.20)	18.71 (0.47)	24.72 (0.53)	50.28 (0.95)	43.82 (1.22)	6.88 (0.24)	18.05 (0.42)	23.04 (0.62)	48.05 (1.05)	38.92 (2.95)

a There was one death in this round that was not considered to be treatment related.

b Dose escalation with the first dose from postnatal day (PND) 4 to PND 21 and the second dose from PND 22 to PND 32.

**FIG 3:**
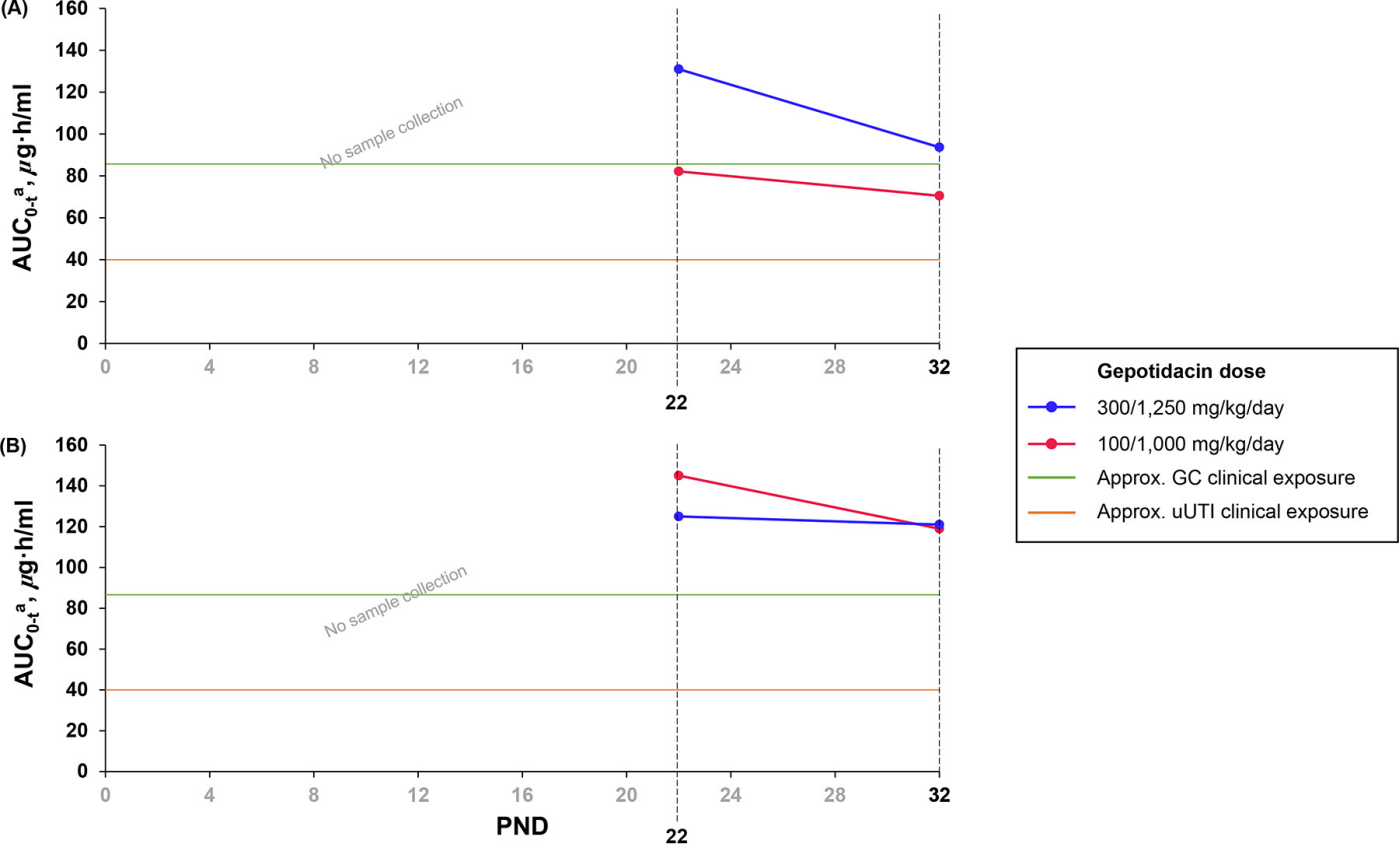
Round 3 toxicokinetics of mean area under the curve from time zero to the last measurable concentration (AUC_0–_*_t_*) values in males (A) and females (B) on postnatal day (PND) 22 and PND 32 by gepotidacin dose with dose escalation on PND 21 to maintain exposure levels and separation between dose groups as much as possible at the potential maximum tolerated doses. The days on which samples were collected are marked in black text. Additional lines show approximate AUC clinical exposures for uncomplicated urinary tract infection (uUTI) and urogenital gonorrhea (GC) indications (40 μg · h/mL [orange] and 85 μg · h/mL [green], respectively). ^a^For the purpose of calculating the AUC_0–_*_t_*, the concentrations at time zero were assigned with the concentrations at 24 h.

In both males and females at doses of 100/1,000 and 300/1,250 mg/kg/day, AUC_0–_*_t_* values decreased slightly from PND 22 to PND 32: males had a larger decrease in the AUC_0–_*_t_* with the 300/1,250-mg/kg/day dose (−37.3 μg · h/mL) than with the 100/1,000-mg/kg/day dose (−11.7 μg · h/mL), while females had a larger decrease in the AUC_0–_*_t_* with the 100/1,000-mg/kg/day dose (−26.0 μg · h/mL) than with the 300/1,250-mg/kg/day dose (−4.0 μg · h/mL). Round 3 AUC_0–_*_t_* values are shown in Fig. 3, and all exposure values are shown in Table S3.

### Macroscopic examination and microscopic evaluation of joints.

No abnormalities were seen in any dose group during the toxicity-and-toxicokinetics phase (rounds 2 and 3) of the study; macroscopic examination of the femoral head and stifle joints from all juvenile male and female rats determined that all joint surfaces were normal.

**FIG 4:**
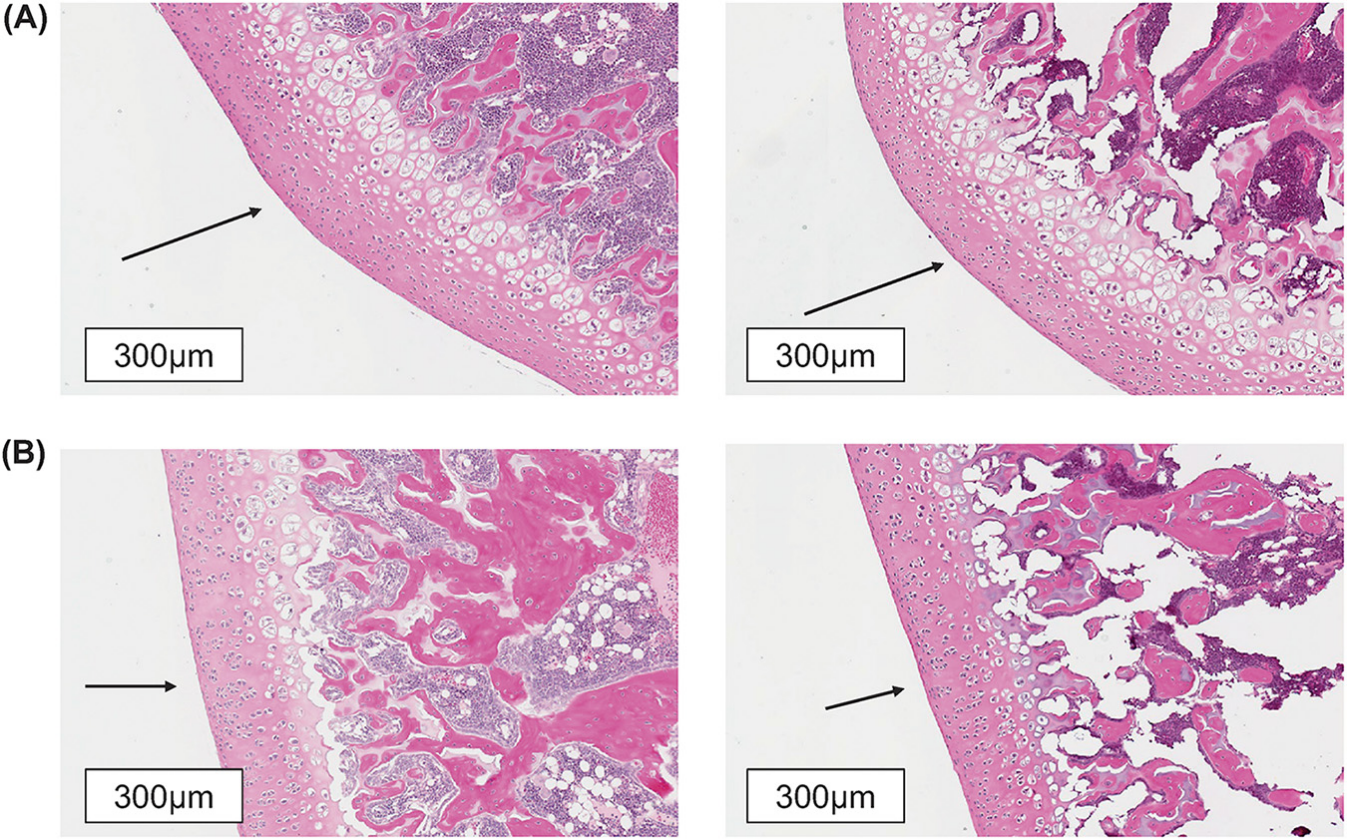
Normal microscopic appearance of articular cartilage of the distal femur (A) and proximal tibia (B) from control juvenile rats (left) and high-dose gepotidacin (300/1,250 mg/kg/day)-treated juvenile rats (right) dosed from postnatal day (PND) 4 to PND 32, with necropsy on PND 33. Arrows indicate articular cartilage. A subtle tiling artifact in the white background (of the top right panel) was contained within the digital image file and considered related to computer stitching of images by the Aperio Scanscope instrument (Leica Biosystems, Deer Park, IL, USA).

The microscopic appearance of the articular cartilage of the stifle joint (distal femur and proximal tibia) in juvenile male and female rats given 0 mg/kg/day (control) and 300/1,250 mg/kg/day from PND 4 to PND 32 (round 3) was within normal limits, with no differences between rats given the vehicle control and those given gepotidacin (Fig. 4).

## DISCUSSION

In this nonclinical study to assess the toxicity and plasma toxicokinetics of gepotidacin in juvenile rats, dose levels of up to 300 mg/kg/day were well tolerated when given from PND 4 to PND 21, while higher dose levels of up to 1,250 mg/kg/day were well tolerated in older juveniles treated from PND 22 to PND 35. Gepotidacin is eliminated mostly unchanged in urine (29), and the dose escalation on PND 22 necessary to maintain systemic exposure during entire study period (PND 4 up to PND 35) was considered to be related mainly to the maturation of renal elimination from low neonatal to adult levels (30). Treatment-related mortalities, clinical signs (loose feces), and decreased mean body weight gains were evident for juvenile rats given 1,000 mg/kg/day from PND 4 to PND 21, resulting in the early termination of the 1,000/1,500-mg/kg/day dose group.

After daily treatment for approximately the first month of life at the maximum tolerated dose levels of 300/1,250 mg/kg/day (with the dose adjusted on PND 22), there were no toxic effects of gepotidacin, including no findings in the articular surfaces of the femoral head (macroscopically) or the stifle joint (macroscopically and microscopically), on juvenile rats. At this dose, the sex-averaged mean AUC_0–_*_t_* values were 252, 128, and 107 μg · h/mL, and the mean *C*_max_ values were 29.2, 18.6, and 14.5 μg/mL, on PND 13, PND 22, and PND 32, respectively. Exposures achieved in juvenile rats were approximately 3-fold above the highest clinical exposures of gepotidacin seen in adults and adolescents ≥12 years of age for the uUTI (AUC of approximately 40 μg · h/mL) and urogenital GC (AUC of approximately 85 μg · h/mL) indications (Fig. 3) (27). No evidence of arthropathy risk in children has been identified with gepotidacin compared to that of fluoroquinolones, which subsequently provides support for ongoing and future clinical studies of gepotidacin in children aged ≥2 years. In the phase III trials of gepotidacin in adults and adolescents, the duration of treatment is a maximum of 5 days (22, 23), a much shorter duration of exposure than the one studied here.

Previous studies have shown that postnatal skeletal growth can be assessed in animal studies because the growth and developmental patterns in laboratory animals and humans are similar (31). Our study was performed using an appropriate species and age range to detect fluoroquinolone-like arthropathy since previous evaluations of short-term exposures of juvenile rats to fluoroquinolones have shown that the period between PND 4 and PND 32 is the one most sensitive to treatment-induced arthropathy (32).

Fluoroquinolone arthropathy in juvenile animals develops in weight-bearing areas of the articular-epiphyseal-cartilage complex (32). The most commonly affected site in the hind leg is the distal femur (16), followed by the proximal tibia (33–35). The femoral head, although a potential site of lesion development, is less commonly affected. Fluoroquinolone arthropathy is described microscopically as being focal with an abrupt onset (35, 36). In fact, studies performed in juvenile rats administered a single dose of ofloxacin before sacrifice/evaluation every 3 h revealed that the cartilage contained no microscopically observable changes at 9 h, and then, abruptly, lesions were observable at 12 h (with no gradual transition) (36). The continuum of the severity of changes in the affected cartilage reportedly includes middle zone degeneration and necrosis of chondrocytes, edema and decreased stainability of the matrix, cleft or cavity formation with surface erosion, and clusters of proliferative chondrocytes around the cavity (33). Importantly, microscopic changes in the absence of macroscopic findings have not been reported, and in the present study, both the stifle joint and femoral head were evaluated across a range of doses up to the maximum tolerated dose in juvenile rats. Furthermore, the most susceptible stifle joint was chosen for detailed microscopic examination at the highest tolerated dose.

One of the potential mechanisms of fluoroquinolone arthropathy in juvenile animal models is a deficiency of magnesium in the cartilage due to the divalent cation-chelating properties of quinolones (16). Magnesium is essential for the biochemistry of cartilage, including integrin receptor signaling, and experimental magnesium deficiency alone causes lesions identical to those of fluoroquinolone arthropathy, with an age sensitivity that overlaps that of fluoroquinolone arthropathy in juvenile animals (16, 37). A recent study has shown that experimental magnesium deficiency was associated with higher incidences of fluoroquinolone-induced lesions (36). The triazaacenaphthylene antibiotic gepotidacin does not share the beta-keto acid structure of fluoroquinolones and their ability to bind magnesium and other divalent cation metals (25); thus, gepotidacin is not considered to have a similar risk of arthropathy related to this potential mechanism of toxicity.

Although several studies in children have identified only a weak association between fluoroquinolone use and arthropathy (14), fluoroquinolones are currently reserved for use in children with life-threatening or difficult-to-treat infections or when other antibiotics are contraindicated because of drug allergy, drug toxicity, or antimicrobial resistance (8, 38). Fluoroquinolone-associated tendinopathy is a rare side effect (0.04 to 0.14%) in older adults, and there has been a black box warning on the labeling of fluoroquinolones for this concern since 2008 (39). Although tendinopathy was not studied in the present study, nonclinical models for fluoroquinolone-associated tendinopathy exist (40). In humans, the Achilles tendon is most often affected by fluoroquinolones, with other load-bearing tendons being affected less commonly. Tendinopathy may be associated with rupture and is often bilateral. Similar to fluoroquinolone-associated arthropathy in juvenile animals, tendinopathy lesions are typified by degeneration without inflammation (41). In contrast to older humans, young age increases the susceptibility to tendinopathy in rats (42, 43). In both humans and rat models, the injury to the articular surface/tendon is acute, beginning within days of drug exposure. However, delayed cases of clinical tendinopathy occurring after 18 months have been reported. The pathogenesis of drug-induced tendinopathy is not fully understood, but proposed mechanisms overlap those for arthropathy and include the impact of load/weight bearing and necrosis of tenocytes with disrupted integrin signaling (39, 41). Therefore, the absence of arthropathy with gepotidacin demonstrated in this study may similarly indicate the absence of a risk of tendinopathy.

Gepotidacin has a unique mechanism of action that distinguishes it from quinolone antibiotics and is currently in phase III development for the investigational treatment of uUTIs in female adolescents aged ≥12 years (22, 23) and urogenital GC in male and female adults (24). The entire nonclinical safety package for gepotidacin, including this nonclinical study in juvenile rats, supports the inclusion of adolescents in these clinical trials and children aged 2 to 11 years in trials for uUTI. Given the increasing rate of antibiotic resistance and gepotidacin’s novel mechanism of action, it is anticipated that gepotidacin will increase the therapeutic options for adult and pediatric patients with these infections and provide a potentially safer option than fluoroquinolones, with a reduced risk of arthropathy.

The juvenile rats in this study were at the most susceptible age, and the highly susceptible stifle joint was evaluated for the development of fluoroquinolone-like arthropathy at margins above clinical gepotidacin exposure. The lack of an arthropathy-inducing fluoroquinolone positive-control group in our study could be considered a limitation; however, as fluoroquinolone arthropathy is well characterized in juvenile rats (31), this omission was not considered to impact the interpretation of the data. Furthermore, we strove to refine our study design by reducing the overall number of animals used, in alignment with International Council for Harmonisation guidelines for the design of juvenile animal studies supporting pediatric drug development (44), and the inclusion of a positive-control group was not considered necessary for our study design given the well-characterized nature of fluoroquinolone arthropathy in juvenile rats. This negative result for fluoroquinolone-like arthropathy in a juvenile rat study differentiates gepotidacin from fluoroquinolones with respect to the decreased arthropathy risk in children.

### Conclusion.

Gepotidacin is an antibacterial medicine with a novel mechanism of action that is being developed for infectious diseases where bacterial antibiotic resistance is a problem. Importantly, gepotidacin is expected to fill an unmet need for efficacy and safety, treating infectious diseases where fluoroquinolones are currently used and where side effects limit their clinical utility in younger children, for whom fluoroquinolone arthropathy is a concern. This nonclinical toxicology study treated juvenile rats during the sensitive age range and at dose levels that attained systemic exposure over clinical exposure and included specific endpoints to detect fluoroquinolone-like arthropathy (macroscopic and microscopic evaluations of the femoral head and/or stifle joint). Gepotidacin did not induce arthropathy in juvenile rats, which provides evidence that gepotidacin is not expected to have risks for fluoroquinolone-like arthropathy in children.

## MATERIALS AND METHODS

### Study design.

**FIG 5:**
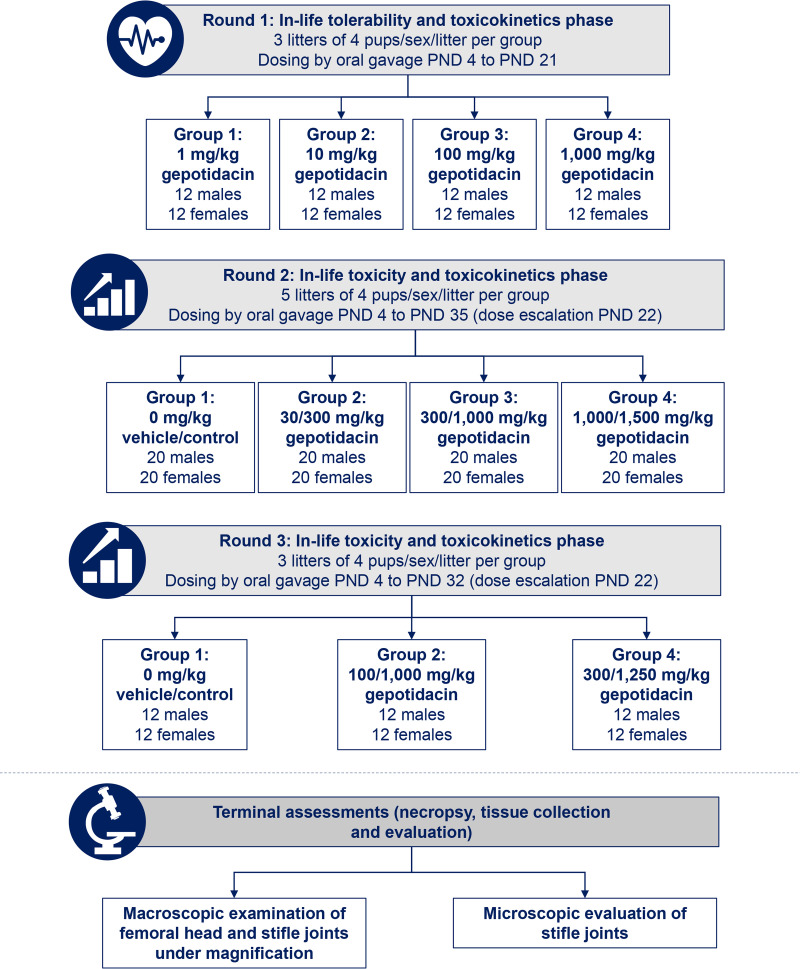
Study design. All rat pups were dosed directly by oral gavage with a gepotidacin suspension (1% aqueous methylcellulose, with a viscosity of 400 cP [2% solution in water]) at a dose volume of 5 mL/kg based on daily body weight. In-life observations included clinical observations and recording of body weight. PND, postnatal day.

This study, with limited focused endpoints to assess potential skeletal effects, was conducted in juvenile male and female Long Evans rats (strain Crl:LE) and was split into two parts: in-life procedures (tolerability and toxicokinetics) and terminal assessments (necropsy, tissue collection, and evaluation); the complete study design is shown in Fig. 5. Good laboratory practice compliance was not required for this study.

Pups derived from 54 10-week-old, time-mated, virus-antibody-free female rats obtained on gestation day 13 or 14 (Charles River Laboratories Inc., Raleigh, NC) were used for in-life procedures. Litters were examined on PND 3 or PND 4 for total pup counts, sex identification, individual weights, and external findings; litters were adjusted to the desired size and sex ratio. No fostering was performed. Dams and litters that were selected for randomization were arbitrarily assigned to study groups based on weight and external examinations (additional details can be found in Text S1 in the supplemental material). Our study consisted of two in-life phases: a tolerability-and-toxicokinetics phase (round 1), to identify tolerated doses and exposures in preweaning rats, and a toxicity-and-toxicokinetics phase (rounds 2 and 3) in pre- and postweaning rats. All rat pups were dosed directly by oral gavage with a gepotidacin suspension (1% aqueous methylcellulose, with a viscosity of 400 cP [when characterized in a 2% solution in water]) at a dose volume of 5 mL/kg based on daily body weight. In-life evaluations focused on clinical signs and body weights.

Terminal assessment procedures included necropsy, tissue collection, macroscopic examination under magnification of the femoral head and femorotibial (stifle) joints, and microscopic evaluation of stifle joints.

All studies were conducted in accordance with the GSK policy on the care, welfare, and treatment of laboratory animals (45) and were reviewed by the Institutional Animal Care and Use Committee at GSK.

**(i) Tolerability-and-toxicokinetics phase (round 1).** Groups of 12 pups of each sex (4 pups/sex/litter [3 litters/group]) were given doses of 1, 10, 100, or 1,000 mg/kg/day once daily on PND 4 to PND 21. A maximum dose of 1,000 mg/kg/day was selected due to the results of a 4-week oral toxicity study in adult rats that showed that a dose of 1,500 mg/kg/day was not tolerated. A wide range of dose levels was selected for this study to ensure that a tolerated dose was attained since juvenile rats may be more or less sensitive than adult animals. Juvenile rats were terminated on PND 13 or PND 21 (the end of the dosing period); toxicokinetics were evaluated on both termination days, and assumed tolerability, based on clinical observations and weight, was assessed throughout the dosing period.

**(ii) Toxicity-and-toxicokinetics phase (round 2 and round 3).** Following large decreases in systemic exposure evident in the tolerability phase (between PND 13 and PND 21 at 100 and 1,000 mg/kg/day), a dose escalation approach was used in an attempt to maintain consistent systemic exposures in all dose groups throughout the treatment period (46).

In round 2, dose levels were 0 (vehicle control), 30/300, 300/1,000, or 1000/1,500 mg/kg/day (lower doses on PND 4 to PND 21 and higher doses on PND 22 to PND 32/35), given once daily from PND 4 to PND 35 to groups of 20 pups (4 pups/sex/litter [5 litters/group]).

Following unexpected mortality in preweaning animals at 1,000 mg/kg/day in round 2 that was not observed in round 1 (the difference may have been due to the larger group size in round 2), for round 3, additional doses of 0 (vehicle control), 100/1,000, or 300/1,250 mg/kg/day were evaluated in groups of 12 pups of each sex (4 pups/sex/litter [3 litters/group]), which were given once daily from PND 4 to PND 32, with dose escalation on PND 22. These additional dose levels were chosen in an attempt to obtain exposure separation between dose groups at the potential maximum tolerated doses.

**(iii) Toxicokinetics.** Blood samples for toxicokinetic evaluation were collected using a composite sampling strategy: nonlittermates, with 1, 2, or 3 pups/sex/time point/group. In round 1, blood samples were collected terminally from the vena cava at 1, 3, 8, and 24 h postdose on PND 13 or from a combination of nonterminal tail snip (1 and 3 h postdose) and terminal vena cava (8 and 24 h) collection procedures on PND 21. Rats were euthanized by carbon dioxide inhalation prior to blood sample collection from the vena cava.

In rounds 2 and 3, blood samples were collected from a combination of tail snip (0, 1, and 3 h postdose) and vena cava (8 and 24 h postdose) collection procedures on PND 22 or PND 32/35. On each sampling occasion, blood samples were spotted onto Whatman FTA (Flinders Technology Associates) cards (dried blood spots), and gepotidacin concentrations were analyzed using high-performance liquid chromatography with mass spectrometry detection. Where data allowed, toxicokinetic analysis of gepotidacin concentration-time data was performed to obtain estimates of the maximum concentration (*C*_max_), time to *C*_max_ (*T*_max_), and area under the curve (AUC) values.

**(iv) Necropsy, tissue collection, and examination.** At study termination in round 2 (PND 35) and round 3 (PND 32), juvenile rats were euthanized, and 6 pups of each sex/group were necropsied to remove eyes, optic nerves, stifle joints, and femoral heads. Care was taken not to damage any internal surfaces of the joint. Soft tissue was sufficiently dissected to ensure that joint surfaces were fully exposed to the fixative. Eyes and optic nerves were fixed in Davidson’s solution after collection and then retained in 10% neutral buffered formalin (NBF). Hind limbs were placed into NBF for ≥48 h prior to macroscopic examination. Following fixation, femoral head and stifle joint surfaces were examined macroscopically under magnification using a dissecting microscope (magnification, ×15 to ×20) to detect alterations in the articular cartilage and were then returned to NBF.

Following a period of storage in the fixative, it was decided to microscopically evaluate the tissues and femur and tibia bones. Stifle joints from rats given the vehicle or gepotidacin at 300/1,250 mg/kg/day in round 3 were placed into Formical-4 decalcifier for ≥72 h and then processed to paraffin blocks; one paraffin block containing the distal femur and one paraffin block containing the proximal tibia (articular surfaces of the stifle joint) were selected for microscopic evaluation. Both blocks included the articular cartilage and were extended to include a portion of the bone diaphysis.

To properly represent the weight-bearing surface of the stifle for both the femur and tibia, two to five microtomy sections from each block per animal were obtained on slides based on articular landmarks. Hematoxylin- and eosin-stained slides for the control and highest-dose groups were examined by a board-certified veterinary anatomic pathologist by light microscopy. Representative original slides from rats given the vehicle or gepotidacin at 300/1,250 mg/kg/day were scanned using an Aperio Scanscope instrument in order to obtain photomicrographs using Imagescope software (both from Leica Biosystems, Deer Park, IL, USA).

### Data availability.

The data supporting the findings of this study are available within the article and its supplemental material. Requests for the data sets generated from the original nonclinical research presented in this article will be considered by the corresponding author upon reasonable request.
