# Supplementary material for: Novel Bacterial Topoisomerase Inhibitor Gepotidacin Demonstrates Absence of Fluoroquinolone-Like Arthropathy in Juvenile Rats
Source: Antimicrob Agents Chemother. 2022 Oct 18;66(11):e00483-22. doi: 10.1128/aac.00483-22 (PMC9664842; doi:10.1128/aac.00483-22)
Supplement: Supplemental file 1 — Supplemental material. Download aac.00483-22-s0001.pdf, PDF file, 0.1 MB [file aac.00483-22-s0001.pdf]

SUPPLEMENTAL MATERIAL

METHODS

Study Design – Additional Detail

During each round of dosing, dams and their litters were selected for randomization into study groups based upon litter weights and external examinations performed on post-natal day (PND) 3 or PND 4. Prior to actual randomization, litters that contained more than 8 pups/litter were reduced to 8 pups/litter, and 4 pups of each sex where possible. No fostering was performed as there were enough litters available to fulfil litter and group size requirements. Furthermore, when pup deaths were found among treated litters on the first or second day of the study, adjustments were made in the number of pups retained from unassigned litters with a later delivery date to ensure that total group size requirements for male and female pups were met. Dams and litters that were determined to be suitable for study assignment (based on litter weight and clinical signs) were arbitrarily selected and assigned to 4 treatment groups (Round 1: 4 pups/sex/litter, 3 litters/group; Round 2: 4 pups/sex/litter, 5 litters/group; Round 3: 4 pups/sex/litter, 3 litters/group). Pups selected for continuation on study were identified as a litter by virtue of their maternal identification number and individually by tattoo markings on the paw. Maternal animals were euthanized at weaning on PND 21 or PND 22 and were discarded without evaluation.

Testing Phase (Round 2 and Round 3)

Pups found dead prior to scheduled euthanasia (excluding cannibalized pups) and pups euthanized due to poor condition, were examined for visceral defects on or prior to Day 7 using a modified Staples Technique; (1, 2) dead pups were also examined for absence of milk in the stomach and evidence of physical trauma associated with the dosing procedure. Unless otherwise noted, juvenile rats were weaned on PND 21 and housed together as a litter by sex until PND 28, and then housed 2 or 3 per box until the end of the study.

REFERENCES

1. Balaji NS, Macklin CP, Fawole S, Aster AS, Rao VS, Moore PJ, Ahmad SM. 2007. The 'modified triple staple' technique: a variant stapling technique for anastomosis after low anterior resection. Surgeon 5:199-201.
2. Ravitch MM, Ong TH, Gazzola L. 1974. A new, precise, and rapid technique of intestinal resection and anastomosis with staples. Surg Gynecol Obstet 139:6-10.

SUPPLEMENTAL TABLE S1 Round 1 toxicokinetic results, by sex and gepotidacin dose

Parameter[a]		Male (*n* = 2/timepoint)				Female (*n* = 1 or 2/timepoint)			
		Gepotidacin dose, mg/kg/day				Gepotidacin dose, mg/kg/day			
		**1**	**10**	**100**	**1000**	**1**	**10**	**100**	**1000**
**AUC_{0-t}[b], μg.h/ml**	**PND 13**	0.460	7.00	70.7	775	NA	9.40	72.7	1010
	**PND 21**	NA	NA	4.47	208	NA	NA	5.64	205
**C_{max}, μg/ml**	**PND 13**	0.078	0.880	9.25	79.2	0.059	1.09	10.1	93.7
	**PND 21**	0.010	0.102	1.36	25.1	NA	0.017	1.80	28.9
**T_{max}, h**	**PND 13**	3	3	3	3	3	1	3	3
	**PND 21**	24	3	3	3	NA	3	3	3

[a]Results reported as composite mean. [b]For the purpose of calculating AUC_{0-t}, the concentrations at time zero were assigned with the concentrations at 24 hours. NA (not applicable) parameters (C_{max} and T_{max}) could not be calculated due to limited measurable data. AUC, area under curve; C_{max}, maximum concentration; PND, post-natal day; T_{max}, time drug is present at maximum concentration.

SUPPLEMENTAL TABLE S2 Round 2 toxicokinetic results, by sex and gepotidacin dose

		Male (n = 2 or 3/timepoint)			Female (n = 1 or 2/timepoint)		
		Gepotidacin dose, mg/kg/day			Gepotidacin dose, mg/kg/day		
**Parameter**[a]		**30/300**[b]	**300/1000**[b]	**1000/1500**[b]	**30/300**[b]	**300/1000**[b]	**1000/1500**[b]
**$AUC_{0–t}$, μg.h/ml**	**PND 13**[c]	19.0	263	NA[d]	21.0	240	NA[d]
	**PND 22**	18.8	94.6	NA[e]	13.5	64.5	NA[e]
	**PND 35**[c]	16.1	105	NA[e]	21.3	122	NA[e]
**C_{max}, μg/ml**	**PND 13**[c]	2.74	30.1	NA[d]	2.67	28.3	NA[d]
	**PND 22**	5.93	10.5	NA[e]	3.73	7.17	NA[e]
	**PND 35**[c]	3.75	12.7	NA[e]	5.96	20.2	NA[e]
**T_{max}, h**	**PND 13**[c]	3	3	NA[d]	3	3	NA[d]
	**PND 22**	3	3	NA[e]	3	3	NA[e]
	**PND 35**[c]	3	8	NA[e]	3	3	NA[e]

[a]Results reported as composite mean. [b]Dose escalation, first dose level given from PND 4 to PND 21, second dose level given from PND 22 to PND 35. [c]For the purpose of calculating $AUC_{0–t}$, the concentration at time zero were assigned with the concentrations at 24 hours. [d]NA: no sample collected due to same dose given on PND 13 in tolerability phase. [e]NA: all animals in the 1000/1500 mg/kg/day group euthanized prior to PND 22. AUC, area under the curve; C_{max}, maximum concentration; NA, not applicable; PND, post-natal day; T_{max}, time drug is present at maximum concentration.

SUPPLEMENTAL TABLE S3 Round 3 toxicokinetic results, by sex and gepotidacin dose

		Male (n = 2 or 3/timepoint)		Female (n = 2 or 3/timepoint)	
		Gepotidacin dose, mg/kg/day		Gepotidacin dose, mg/kg/day	
**Parameter**[a]		**100/1000**[b]	**300/1250**[b]	**100/1000**[b]	**300/1250**[b]
**$AUC_{0–t}$, μg.h/ml**	**PND 22**	82.2	131	145	125
	**PND 32**[c]	70.5	93.7	119	121
**C_{max}, μg/ml**	**PND 22**	16.8	16.2	19.2	21.0
	**PND 32**[c]	8.43	11.4	15.1	17.6
**T_{max}, h**	**PND 22**	3	3	3	3
	**PND 32**[c]	8	3	8	3

[a]Results reported as composite mean. [b]Dose escalation, first dose level given from PND 4 to PND 21, second dose level given from PND 22 to PND 32. [c]For the purposes of calculating $AUC_{0–t}$, the concentrations at time zero were assigned with the concentrations at 24 hours. AUC, area under the curve; C_{max}, maximum concentration; PND, post-natal day; T_{max}, time drug is present at maximum concentration.
